# *NRF2 -617 C/A* Polymorphism Impacts Proinflammatory Cytokine Levels, Survival, and Transplant-Related Mortality After Hematopoietic Stem Cell Transplantation in Adult Patients Receiving Busulfan-Based Conditioning Regimens

**DOI:** 10.3389/fphar.2020.563321

**Published:** 2020-12-15

**Authors:** Jingjing Huang, Chenxia Hao, Ziwei Li, Ling Wang, Jieling Jiang, Wei Tang, Lining Wang, Weixia Zhang, Jiong Hu, Wanhua Yang

**Affiliations:** ^1^ Department of Pharmacy, Ruijin Hospital, Shanghai Jiao Tong University School of Medicine, Shanghai, China; ^2^ Shanghai Jiao Tong University School of Medicine, Shanghai, China; ^3^ Department of Hematology, Ruijin Hospital, Shanghai Jiao Tong University School of Medicine, Shanghai, China

**Keywords:** busulfan, polymorphism, proinflammatory, transplant-related mortality, NF-E2-related factor 2 (Nrf2), hematopoietic stem cell transplantation

## Abstract

Busulfan (BU) is widely used in conditioning regimens prior to hematopoietic stem cell transplantation (HSCT). The exposure-escalated BU directed by therapeutic drug monitoring (TDM) is extremely necessary for the patients with high-risk hematologic malignancies in order to diminish relapse, but it increases the risk of drug-induced toxicity. BU exposure, involved in the glutathione- (GSH-) glutathione S-transferases (GSTs) pathway and proinflammatory response, is associated with clinical outcomes after HSCT. However, the expression of genes in the GSH-GSTs pathway is regulated by NF-E2-related factor 2 (Nrf2) that can also alleviate inflammation. In this study, we evaluated the influence of *NRF2* polymorphisms on BU exposure, proinflammatory cytokine levels, and clinical outcomes in HSCT patients. A total of 87 Chinese adult patients receiving twice-daily intravenous BU were enrolled. Compared with the patients carrying wild genotypes, those with *NRF2 -617 CA/AA* genotypes showed higher plasma interleukin (IL)-6, IL-8 and tumor necrosis factor (TNF)-α levels, poorer overall survival (OS; RR = 3.91), and increased transplant-related mortality (TRM; HR = 4.17). High BU exposure [area under the concentration-time curve (AUC) > 9.27 mg/L × h)] was related to BU toxicities. Furthermore, *NRF2 -617 CA/AA* genotypes could significantly impact TRM (HR = 4.04; *p* = 0.0142) and OS (HR = 3.69; *p* = 0.0272) in the patients with high BU AUC. *In vitro*, we found that high exposure of endothelial cell (EC) to BU, in the absence of Nrf2, elicited the hyperstimulation of NF-κB-p65, accompanied with the elevated secretion of proinflammatory cytokines, and led to EC death. These results showed that *NRF2 -617 CA/AA* genotypes, correlated with high proinflammatory cytokine levels, could predict inferior outcomes in HSCT patients with high BU AUC. Thus, *NRF2 -617 CA/AA* genotyping combined with TDM would further optimize personalized BU dosing for sufficient efficacy and safety endpoint.

## Introduction

The bifunctional alkylating agent busulfan (1, 4-butanediol dimethanesulfonate; BU) is widely used as a major component of conditioning regimens prior to hematopoietic stem cell transplantation (HSCT) (Bredeson et al., 2013). BU exposure, which is expressed as area under the plasma concentration-time curve (AUC), has a narrow therapeutic range of 7.38–13.3 mg/L × h (BU at 1.6 mg/kg for twice-daily dosing) and is significantly correlated with the clinical outcomes: subtherapeutic AUC leads to relapse or graft failure, whereas the supratherapeutic BU AUC results in the fatal toxicity such as hepatic sinusoidal obstruction syndrome (SOS) (Andersson et al., 2017; Philippe et al., 2019; Hill et al., 2020). Personalized BU dosing, directed by therapeutic drug monitoring (TDM), can optimize the target BU exposure to improve clinical outcomes (Palmer et al., 2016). It is necessary for the patients with high-risk hematologic malignancies to achieve the target BU of 28.7 mg/L × h (daily dosing) in order to diminish relapse (Shea et al., 2015). However, the escalation of BU exposure probably induces the risk of SOS (O’Donnell et al., 2010). Previous studies showed interindividual variability in BU exposure, which was associated with different blood glutathione (GSH) and glutathione S-transferases (GSTs) such as GSTA1 and GSTA2 (Almog et al., 2011; Bonifazi et al., 2014; Yin et al., 2015). BU is metabolized mainly in liver through GSTs, a family of phase II detoxification enzymes that catalyze the conjugation of GSH to various xenobiotics (Gibbs et al., 1996).

It is reported that hepatic depletion of GSH contributing to oxidative stress and proinflammatory response provoke BU toxicity (Nagai et al., 2002; Dressel et al., 2003; Jones et al., 2007; Qiao et al., 2015). The GSH-GSTs pathway for BU includes GSTs, glutamate cysteine ligase (GCL) regulating GSH synthetase, and multidrug resistance-associated proteins (MRPs) which transport GSH conjugates across the cell membrane. The expressions of genes in this pathway are manipulated by NF-E2-related factor 2 (Nrf2). Nrf2, a cellular sensor for exogenous oxidative and toxic stress (Nguyen et al., 2009), has been known to attenuate inflammation (Kobayashi et al., 2016). Interestingly, Bouligand et al., 2016, recorded a significant elevation in GSTA1, GSTA2, and glutamate cysteine ligase catalytic subunit (GCLC) transcripts after the fifth BU injection in a mice experiment. Based on this, we hypothesize that BU activates Nrf2 transcription. Genetic polymorphisms, which are involved in drug metabolism and targeting, respectively influence PK and pharmacodynamics of a specific drug (Evans and Johnson., 2001). *NRF2* -617 *C/A* polymorphism is located at the antioxidant-response-element (ARE-) like site that mediates transcriptional responses and exhibits key regulatory roles in various cellular responses after oxidant and toxic insults (Marzec et al., 2007). Thus, the SNP of *NRF2* -617 C/A, which decreases the basal Nrf2 protein level, may impair the GSH-GSTs activity and anti-inflammation action.

In this study, we evaluated the influence of genetic polymorphisms related to the GSH-GSTs pathway such as Nrf2, on BU exposure, proinflammatory cytokine levels, transplant-related mortality (TRM), and overall survival (OS) in Chinese adult patients receiving HSCT.

## Materials and Methods

### Patients

#### Enrollment

Adult patients who underwent a first allo-HSCT for malignant diseases were enrolled at the blood marrow transplantation ward in the Department of Hematology, Ruijin Hospital, Shanghai, China, from July 2011 to December 2016. The exclusion subjects included those aged <18 years or ≥65 years, those who had received BU treatment for <4 days, and those who were lost to follow-up or lacked outcome data. This study was approved by the Research Ethics Committee of Ruijin Hospital, Shanghai, China (No. 2011-016). Written informed consents were obtained from all the enrolled patients.

#### Conditioning, Graft-versus-Host Disease (GVHD) and SOS Prophylaxis, and Supportive Care

All patients received intravenous BU over a 2-h period at the dosage of 1.6 mg kg^−1^ twice daily for 4 days. BU, combined with cyclophosphamide (CY) or fludarabine (FLU), was administered to the patients as a part of a myeloablative regimen. The day of stem cell transplantation is routinely defined as day 0. The BU/CY regimen included BU from day −7 to −4 and CY at the dosage of 60 mg kg^−1^ daily on day −3 and day −2. The BU/FLU regimen was administered, with FLU at 30 mg m^−2^ daily following 4 days of BU from day −6 to day −3. The antithymocyte globulin (ATG) was intravenously infused at the dosage of 2.5 mg/kg on day −2 and day −1 for the BU/CY + ATG regimen or BU/FLU + ATG regimen. CY was administered intravenously at the dosage of 50 mg kg^−1^ daily on days 3 and 4 for the BU-FLU-CY regimen. The dosing of BU, CY, and ATG was provided according to the actual body weight or the adjusted ideal body weight (AIBW) when the patient’s body mass index was >30 kg/m^2^ (Ruutu et al., 2019).

The prophylaxis of GVHD was followed by treatment with cyclosporine A (CsA) plus short-term methotrexate (MTX). Intravenous cyclosporine A (CsA) was administered at the dosage of 1.5 mg/kg every 12 h from day −1. Once the gastrointestinal symptoms improved, 5 mg/kg of CsA was administered orally once daily. The CsA trough level was monitored to target the optimal range of 200–250 ng/ml (Park et al., 2016). Next, 15 mg/m^2^ of MTX was administered on day 1 and that of 10 mg/m^2^ on day 3 and day 6. For patients undergoing haploidentical sibling donor transplantation and unrelated donor transplantation, mycophenolate mofetil (MMF) was administered orally at the dosage of 1.0 g once daily from the beginning of conditioning regimen. Next, the dose of MMF was reduced to 0.5 g once daily or adjusted according to individual condition. The specific treatments for GVHD prophylaxis, which impact plasma cytokine levels, were not used in the enrolled patients. For hepatic SOS prophylaxis, lipo-prostaglandin E1 was used at the dosage of 0.5 ug/kg once daily from day −7 to day 30. All patients received oral phenytoin at the dosage of 100 mg twice daily for seizure prophylaxis from the day before the initiation of BU infusion to day 0, while oral metronidazole therapy was initiated at the dosage of 200 mg thrice daily and not administered simultaneously with BU on the first day of hospital admission for intestinal decontamination.

#### Blood Sampling and Determination

Blood samples were collected before BU infusion and at 0.5, 1, 2, 3, 4, 5, 6, 8, 10, and 12 h for full sampling strategy or at 2, 3, and 6 h for limited sampling scheme after the infusion of the seventh dose. Whole blood samples were drawn from the peripheral vein in the arm opposite to the central line for busulfan infusion. The whole blood sample (3 ml) was then collected in EDTA-anticoagulant glass tubes. 1 ml of whole blood before BU infusion was for DNA extraction. The plasma of BU was separated from the residual whole blood after centrifugation at 3,000 rpm for 10 min. The whole blood and plasma samples were stored at −80 °C. The BU plasma concentrations were determined with a validated analytical method (Huang et al., 2019) by liquid chromatography electrospray tandem mass spectrometry.

#### DNA Extraction and Genetic Analysis

DNA was extracted from peripheral blood lymphocytes (Kim et al., 2011) by TIANamp Blood DNA Kit (Tiangen Biotech Co., Ltd, Beijing, China). Genetic polymorphisms related to BU-GSH metabolism were analyzed, including SNPs in NRF2, GSTA1, GSTA2, GSP1, GCLC, GCLM, MRP1, and MRP2. These genotypes were analyzed by SNaPshot assay as per the manufacturer’s protocols (ABI SNaPshot Multiplex kit, CA, USA), shown in Supplementary Table S1.

#### Estimation of BU Exposure by Population Pharmacokinetic Analysis

The population pharmacokinetic (PPK) model of intravenous BU was established (as shown in the Supplementary Data Sheet 1) to estimate BU AUCs by numerical integration using nonlinear mixed-effect model (NONMEM) methodologies (Bartelink et al., 2016).

## Outcomes Evaluation

### Plasma Proinflammatory Cytokine Level Monitoring

Plasma proinflammatory cytokine levels, including interleukin (IL)-6, IL-8, and tumor necrosis factor (TNF)-α, were monitored from day -8 to day 34. Venous blood samples were collected with EDTA anticoagulation between 07:00 AM and 09:00 AM once daily on day -8 and from day 1 to day 14. Then, other samples were obtained every 5 days from day 15 to day 34. All samples were centrifuged at 3,000 rpm for 10 min and the plasma was extracted and determined by enzyme-linked immunosorbent assay (ELISA) kits (Elabscience Biotechnology Co., Ltd., Wuhan, China).

#### Complications, TRM, and OS

The evaluated complications after HSCT included disease relapse or graft-failure (defined as nonengraftment or rejection) associated with subtherapeutic BU AUC (<7.38 mg/L × h) and the toxicities: veno-occlusive disease (VOD)/SOS as per Bearman (Bearman, 1995), acute GVHD (aGVHD) grade II-IV as per Glucksberg (Glucksberg et al., 1974), and chronic GVHD (cGVHD) as per the Shulman criteria (Shulman et al., 1980). The association between the toxicities and high BU AUC was analyzed by receiver operating characteristic (ROC) analysis. The primary outcome was TRM defined as death unrelated to any underlying disease. Incidences of relapse and OS were also observed. All surviving patients were censored at the last follow-up day. The duration of followup was from the time of allo-HSCT to that of the last assessment for surviving patients or death.

### *In Vitro* Experiments

#### Chemicals, Reagents, and Antibodies

Dulbecco’s modified Eagle’s medium (DMEM), fetal bovine serum (FBS), and penicillin-streptomycin-neomycin were obtained from Jiancheng Bioengineering Institute (Nanjing, China). Nfe212 Stealth siRNAs, Stealth RNAi Negative Control Duplexes, Opti-MEM, Lipofectamine 2000, and TRIzol for RNA extraction were acquired from Invitrogen (Carlsbad, CA, USA). All primers for PCR were provided by Biological Engineering Technology. First-strand cDNA synthesis kits and SYBR Green Quantitative Kits for real-time PCR were purchased from Fermentas (USA). MTT Cell Proliferation and Cytotoxicity Assay Kit and Annexin V- Fluorescein Isothiocyanate (FITC)/Propidium Iodide (PI) Apoptosis Detection Kit were acquired from Gefan Biotechnology Co., Ltd (Shanghai, China). Nuclear and Cytoplasmic Protein Extraction Kit was obtained from Beyotime Biotechnology (Shanghai, China). Primary antibodies against Nrf2, nuclear factor-κb (NF-κB) p65, Kelch-like ECH associated protein 1 (Keap1), Cyclooxygenase-2 (Cox2), TNF-α, IL-1β, β-Actin, and horse radish peroxidase (HRP)-goat anti-rabbit IgG were purchased from Cell Signaling Technology (USA), whereas antibodies against Heme Oxygenase 1 (HO-1), NQO1, GCLC, and inducible nitric oxide synthase (iNOS) were obtained from Abcam Inc. (Cambridge, MA). All other chemicals and reagents were of analytical grade.

#### Cell Culture and Nrf2 Knockdown by siRNA Transfection

Human vascular endothelial cell lines (EA. hy 926) were purchased from Shanghai Institutes for Biological Sciences, Chinese Academy of Cell Resource Center (Shanghai, China). The cells were grown in DMEM supplemented with 10% FBS, 1% glutamine, and 1% penicillin-streptomycin-neomycin at 37 °C under 5% CO_2_ humidified incubation condition. NRF2-specific siRNA and GC-matched control siRNA were transfected by Lipofectamine 2000 (Life Technologies) to EA. hy 926 cells (at a density of 2.5 × 10^5^ cells/well in a 6-well plate for 48 h).

#### Cell Viability and Apoptosis Assay

EA. hy 926 cells were cultured in 96-well plates, preincubated with BU (5–200 μmol), and allowed to proliferate for 24 h. The cell viability treated by BU was monitored by the MTT reduction assay. Apoptosis was detected using the Annexin V-FITC/PI detection kit as per the manufacturer’s protocol. Briefly, the cells were collected, washed thrice with cold phosphate buffered saline (PBS), and gently suspended in 400-μl binding buffer, followed by staining with Annexin V- FITC (5 μl) and PI (10 μl) solution and incubating for 15 min for analysis by flow cytometry (BD FACS Canto™).

#### Real-Time PCR Analysis

Total RNA was extracted from cells with TRIZOL reagent. cDNA was synthesized and real-time PCR was performed. The relative expression of target genes was standardized to GAPDH, as evaluated by the 2^−∆∆Ct^ method and expressed as a ratio to control. The primer sequences are listed in Supplementary Table S2.

#### Western Blot Analysis

EA. hy 926 cells of control and siNRF2 were inoculated at a density of 2.5 × 10^5^ cells/well into a 6-well plate, followed by preincubation with BU (0–200 μmol) for 24 h. The cytoplasmic and nuclear proteins (Nrf2 and NF-κB p65) were extracted by using a nuclear and cytoplasmic protein extraction kit, respectively, according to the manufacturer’s protocol. The cytoplasmic or nuclear proteins were analyzed by sodium dodecyl sulphate-polyacrylamide gel electrophoresis (SDS-PAGE) and transferred onto transmembrane to probe with specific antibodies. Immunoreactive polypeptides were detected by the chemiluminescent detection system (Alpha Fluor Chem FC3, USA). Image acquisition and analysis were performed by using ImageJ software. The obtained data were adjusted to Lamin B1 or β-actin expression and expressed as a percent of control required to eliminate variations resulting from protein quantity and quality. All experiments were performed independently at least thrice.

#### Statistical Analysis

The continuous variables were represented as mean with standard deviation or 95% CIs. The statistical significance of the difference between the groups was calculated by two one-sided t-tests, one-way analysis of variance (ANOVA) with least significant difference (LSD) methods, or Chi-square test. Histograms of plasma proinflammatory IL-6, IL-8, and TNF-α levels were analyzed with the locally weighted scatter plot smoothing curves. The competing risk analysis (univariate analysis) was adopted for TRM. Multivariate analysis was performed by the Fine-Grey model (competing risk regression) for TRM and the Cox proportional hazards model for OS and relapse. The *p* values of multivariate analysis were adjusted by Benjamini-Hochberg correction.

The above analysis was performed by the R software package (version 3.6.3). The incidences of TRM, OS, and relapse were analyzed by Kaplan-Meier curves using GraphPad Prism software (GraphPad Software Inc., La Jolla, California, USA). The *p* values <0.05 were considered to be statistically significant.

## Results

### Patient Characteristics, Genetic Frequencies, and BU Exposure

A total of 87 adult patients [mean age: 32.9 ± 11.1 years; 66.7% (58/87) men] were enrolled in this study, of which 62 (71.3%) patients had the disease status of complete remission (CR) before HSCT (Table 1). Eighty-five (97.7%) patients were transplanted by matched sibling donors or matched unrelated donors. In addition, 32 (36.8%) patients received BU/CY-based conditioning and 55 (63.2%) received BU/FLU-based conditioning. The frequencies of 16 genetic polymorphisms are shown in Supplementary Table S3. There were 37 patients with *NRF2 -617CA/AA* genotypes and 50 with wild genotype. Moreover, the frequency of *NRF2 -617 A* allele here was 24.7% (Asians: 24.3%), greater than those in the Europeans (12.5%) and Southern Asians (15.2%). All genetic frequency distributions were in Hardy-Weinberg equilibrium. These minor allele frequencies were found to be similar to those from East Asians.

**TABLE 1:** Demographic and baseline characteristics of the enrolled patients.

Variables		Values
Number of patients		87
Age (years)		32.9 ± 11.1
Male, n (%)		58 (66.7)
Weight (kg)		63.2 ± 12.9
Height (cm)		169 ± 7.98
BSA (m^2^)		1.72 ± 0.186
BMI (kg/m^2^)		22.1 ± 3.93
	Underweight (<18.5)	14 (16.1)
	Normal (18.5–24.9)	56 (64.4)
	Overweight (25.0–29.9)	14 (16.1)
	Obese (>30)	3 (3.4)
Hepatic function	AST (U/L)	22.5 ± 10.9
	ALT (U/L)	33.0 ± 29.8
	ALP (U/L)	63.4 ± 27.5
	TBIL (μmol/L)	12.5 ± 5.94
Diagnosis, *n* (%)	ALL	23 (26.4)
	AML	43 (49.4)
	CML	6 (6.9)
	MDS	7 (8.0)
	HL	2 (2.4)
	NHL	6 (6.9)
Disease status at transplantation, *n* (%)	CR	62 (71.3)
	PR	16 (18.4)
	Active disease	3 (3.4)
	Untreated	6 (6.9)
Type of donor, *n* (%)	Matched sibling donor	40 (46.0)
	Haploidentical sibling donor	2 (2.3)
	Matched unrelated donor	45 (51.7)
Conditioning regimen, *n* (%)	BU/CY	17 (19.5)
	BU/CY + ATG	15 (17.3)
	BU/FLU	23 (26.4)
	BU/FLU + ATG	17 (19.5)
	BU/FLU + CY	15 (17.3)
GVHD prophylaxis, *n* (%)	CSA + MTX	57 (65.5)
	CSA + MTX + MMF	30 (34.5)

ALL, acute lymphoblastic leukemia; ALP, alkaline phosphatase; ALT, alanine aminotransferase; AML, acute myelocytic leukemia; AST, aspartate transaminase; ATG, antithymocyte globulin; BMI, body mass index according to National Heart, Lung, and Blood Institute weight categories; BSA, body surface area; CML, chronic myelocytic leukemia; CY, cyclophosphamide; CR, complete remission; CsA, cyclosporine A; FLU, fludarabine; GVHD, graft vs. host disease; HL, Hodgkin's lymphoma; MDS, myelodysplastic syndromes; MMF, mycophenolate mofetil; MTX, methotrexate; NHL, non-Hodgkin lymphoma; PR, partial remission; TBIL, total bilirubin.

A total of 760 BU concentrations from 87 patients were obtained for PPK modeling (Supplemental PPK data, Supplementary Figures S1,S2). The mean AUC of dose seven was 8.54 mg/L × h (95% CI: 8.06–9.02 mg/L × h) in Chinese HSCT patients. Of all, 75 (86.2%) patients had above therapeutic AUC (>7.38 mg/L × h), including 5 with supratherapeutic AUC (>13.3 mg/L × h). Moreover, PPK analysis revealed that conditioning regimen and BSA were significant covariates on BU CL and Vd, respectively. However, no genetic polymorphisms significantly impacted BU CL.

### Clinical Outcomes: NRF2 -617 C/A Polymorphism Influenced Plasma Proinflammatory Cytokine Levels, TRM, and OS

#### The Proinflammatory Cytokine Levels Were Elevated During the Early Phase After HSCT in Adult Patients With NRF2 -617 CA/AA Genotypes

**FIGURE 1:**
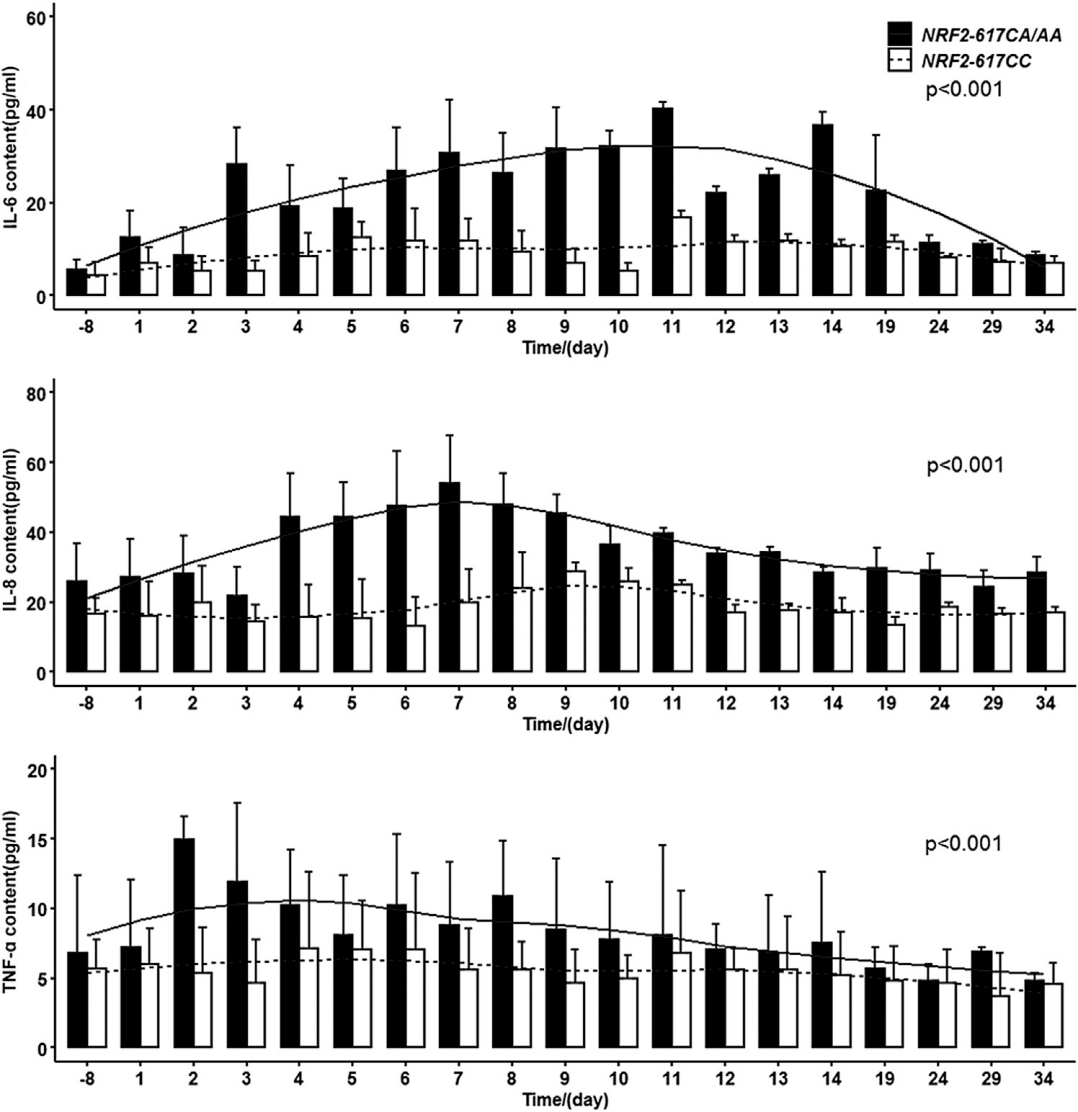
Higher plasma IL-6, IL-8, and TNF-α levels after HSCT in adult patients with *NRF2 -617 CA/AA* genotypes (*n* = 37) compared to those with wild genotype (*n* = 50). Histograms of IL-6, IL-8, and TNF-α levels in the patients with *NRF2 -617 CA/AA* genotypes and *NRF2 -617 CC* genotype. Solid and dotted curves represent the locally weighted scatter plot smoothing curves.

Higher plasma IL-6, IL-8, and TNF-α levels were observed in the patients with *NRF2 -617CA/AA* genotypes (*n* = 37) compared to those with wild genotype (*n* = 50) [GLM estimated marginal means: 22.1 ± 10.2 vs. 9.12 ± 3.21 pg/ml, F = 27.799, *p* < 0.001 (IL-6); 35.3 ± 9.45 vs. 18.6 ± 4.35 pg/ml, F = 49.309, *p* < 0.001 (IL-8); 8.26 ± 2.50 vs. 5.50 ± 0.960 pg/ml, F = 20.083, *p* < 0.001 (TNF-α)], as shown in Figure 1. The IL-6 levels increased significantly from days 3 to 14 after transplantation relative to the levels before busulfan treatment [peak (day 11): 40.3 ± 3.39 pg/ml vs. baseline: 5.70 ± 1.97 pg/ml; *p* = 0.028]. The IL-8 levels were elevated between days 4 and 9 [peak (day 7): 53.9 ± 13.9 pg/ml vs. baseline: 26.0 ± 10.8 pg/ml; *p* = 0.002]. The TNF-α significantly increased on days 3 and 4 [11.9 ± 5.67 pg/ml and 10.2 ± 4.01 pg/ml vs. baseline: 6.84 ± 5.50 pg/ml; *p* = 0.002 and *p* = 0.013]. In addition, the other genetic polymorphisms, conditionings (BU/FLU or BU/CY), and BU AUC (subtherapeutic or above-therapeutic) were not correlated with the proinflammatory cytokine levels (Supplementary Table S4).

#### NRF2 -617 CA/AA Genotypes Significantly Predicted High TRM and Poor OS in Adult Patients With High BU AUC

**FIGURE 2:**
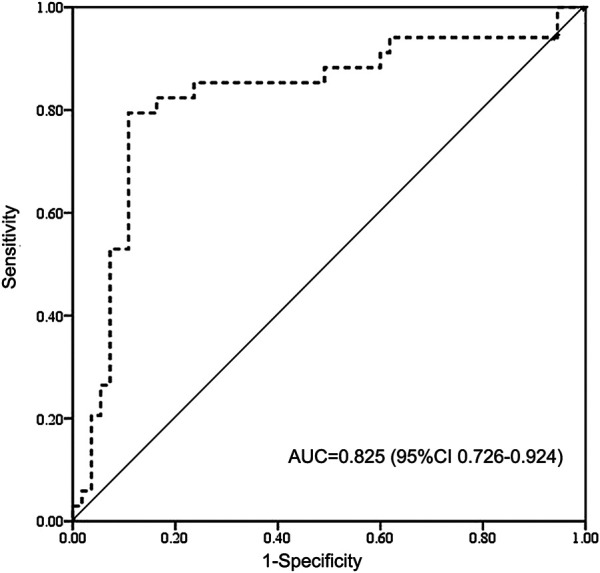
Receiver operating characteristic (ROC) curve of high BU exposure for SOS and GVHD. BU AUC of 9.27 mg/L × h cut point indicates 85% sensitivity and 71% specificity on high-exposure-related toxicity. BU AUC, area under the concentration-time curve of busulfan; AUC, area under the curve for ROC curve; CI, confidence interval; GVHD, graft vs. host disease; SOS, sinusoidal obstruction syndrome.

The results of complications after HSCT were shown in Table 2. The probability of VOD/SOS and aGVHD was 5.7% and 16.1% including 11.5% of aGVHD II and 4.6% of aGVHD III-IV. Two (2.3%) patients had cGVHD at 4 and 9 months after HSCT. The probabilities of relapse and graft failure were 10.6% and 4.6%, respectively. The patients with above therapeutic AUC mainly suffered acute toxicity, including aGVHD (17.3%, 13/75) or VOD (6.7%, 5/75), while half of the patients with subtherapeutic exposure experienced relapse (41.6%, 5/12) or graft failure (8.3%, 1/12). ROC analysis revealed that high BU AUC (>9.27 mg/L × h) was related with SOS and GVHD (Figure 2).

**TABLE 2:** Complications after HSCT.

Complications	Patients with subtherapeutic BU exposure (*n* = 12)	Patients with above therapeutic BU exposure (*n* = 75)	Total
Relapse, *n* (%)	5 (41.6)	4 (5.3)	9 (10.6)
Graft failure, *n* (%)	1 (8.3)	3 (4)	4 (4.6)
VOD/SOS, *n* (%)	0 (0)	5 (6.7)	5 (5.7)
Mild	0 (0)	1 (1.3)	1 (1.1)
Moderate	0 (0)	3 (4)	3 (3.4)
Severe	0 (0)	1 (1.3)	1 (1.1)
aGVHD, *n* (%)	1 (8.3)	13 (17.3)	14 (16.1)
II	1 (8.3)	9 (12)	10 (11.5)
III-IV	0 (0)	4 (5.3)	4 (4.6)
cGVHD, *n* (%)	0 (0)	2 (2.7)	2 (2.3)

aGVHD, acute graft-versus-host disease; cGVHD, chronic graft-versus-host disease; VOD, veno-occlusive disease; SOS, sinusoidal obstruction syndrome.

With a median followup of 18 (1–59) months, the cumulative TRM was 24.3%. The OS was 71.5%. Univariate analysis suggested the impact of the involved genetic polymorphisms on incidences of relapse, TRM, and OS (Supplementary Table S5). Multivariate analysis revealed that BU AUC was correlated with the risk of TRM (high AUC > 9.27 mg/L × h, *n* = 35) and relapse (subtherapeutic AUC< 7.38 mg/L × h, *n* = 12) with HR = 3.94, *p* = 0.048 and RR = 2.42, and *p* = 0.005, respectively. Interestingly, the patients with *NRF2 -617* CA/AA genotypes showed high TRM and poor OS (*p* = 0.041 and *p* = 0.043; Table 3).

**TABLE 3:** Multivariable analysis on TRM, OS, and relapse.

Variables	TRM			OS			Relapse		
	HR	95% CI	*p*	HR	95% CI	*p*	HR	95% CI	*p*
Age	1.04	0.979–1.10	0.263	1.02	0.976–1.066	0.377	1.04	0.989–1.03	0.385
Disease status at transplantation (CR vs. others)	0.503	0.180–1.41	0.263	0.417	0.163–1.066	0.140	0.667	0.398–1.118	0.207
Conditioning regimen (BUCY vs. BUFLU)	1.57	0.531–4.65	0.410	1.65	0.633–4.31	0.195	1.12	0.650–1.94	0.681
BU AUC*	3.94	1.25–12.4	0.048	2.71	0.89–8.23	0.105	2.42	1.45–4.06	0.005
*NRF2 -617 CA/AA*	4.17	1.44–12.0	0.041	3.91	1.19–7.63	0.040	1.58	0.962–2.61	0.178

BU, busulfan; CR, complete remission; CY, cyclophosphamide; FLU, fludarabine; OS, overall survival; TRM, transplant-related mortality; * high busulfan AUC (>9.27 mg/L × h) or not for TRM and OS; subtherapeutic AUC (<7.38 mg/L × h) or not for relapse.

**FIGURE 3:**
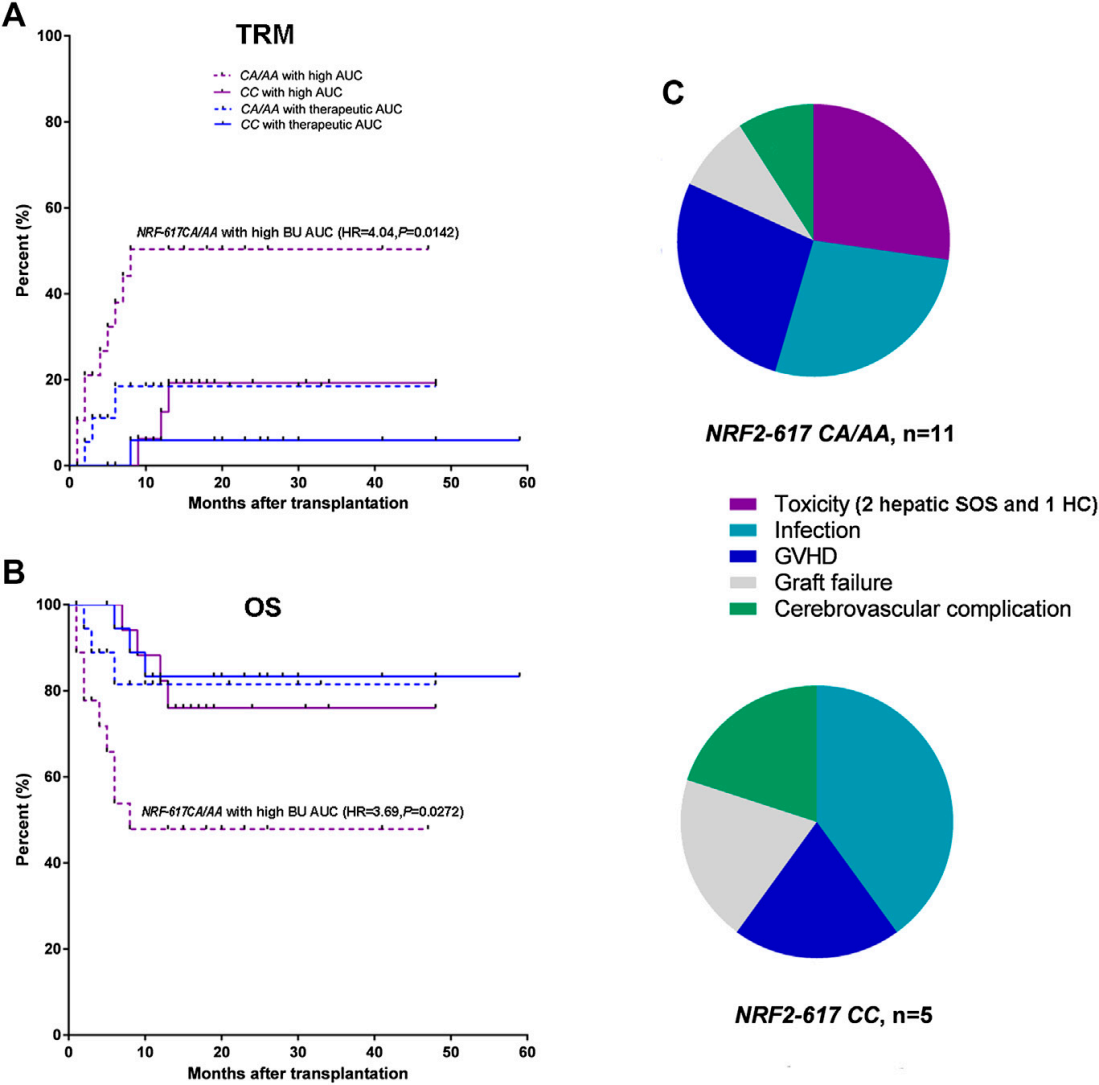
*NRF2 -617 CA/AA* genotypes predicted high TRM and poor OS in adult patients with above therapeutic AUC (*n* = 75). **(A)** Kaplan-Meyer curves of TRM and **(B)** OS according to BU AUC and *NRF2 -617C/A* genotypes. Blue and red lines represent the mortality or survival curves for the patients with therapeutic AUC (*n* = 40) and with high AUC (*n* = 35). Solid and dotted lines represent the curves for the patients with wild type (*CC*) and with heterozygous or homozygous genotype (*CA*/*AA*)*.* (*AA*/*CA* in the patients with therapeutic AUC, *n* = 19; *CC* in the patients with therapeutic AUC, *n* = 21; *AA/CA* in the patients with high AUC, *n* = 19; *CC* in the patients with high AUC, *n* = 16) **(C)** Pie-chart representation of TRM causes of death in the patients with *NRF2 -617CA/AA* (*n* = 11) and *NRF2 -617CC* (*n* = 5). The toxicities were hepatic SOS (2 patients) and hemorrhagic cystitis (1 patients).

Furthermore, Kaplan-Meier estimates were performed to analyze the influence of *NRF2 -617C/A* polymorphism on TRM and OS in the patients with above therapeutic AUC (*n* = 75). The relapse was not a competing risk event for TRM to analyze the influence of *NRF2 -617C/A* polymorphism (TRM: *p* = 0.01; relapse: *p* = 0.66). It was highlighted that *NRF2 -617C/A* polymorphism significantly impacted TRM and OS in patients with high BU AUC [*n* = 35; CA/AA (*n* = 19) vs. CC(*n* = 16); TRM: HR = 4.04, *p* = 0.0142; OS: HR = 3.69, *p* = 0.0272] compared to those with therapeutic AUC [*n* = 40; CA/AA (n = 19) vs. CC(n = 21); TRM: HR = 2.21, *p* = 0.365; OS: HR = 1.62, *p* = 0.462], as shown in Figure 3A,B. The analysis of causes of TRM death showed a higher number of deaths due to conditioning-related toxicity in *NRF2 -617 CA/AA* patients (6 of 11, 54.5%), compared to *NRF2 -617CC* (1 of 5, 20.0%) (Figure 3C).

### *In Vitro* Experiment: High Exposure of Endothelial Cell to BU-Induced EC Death and a Proinflammatory Response in the Absence of Nrf2

#### High Exposure of EC to BU-Induced EC Death and a Proinflammatory Response in the Absence of Nrf2

**FIGURE 4:**
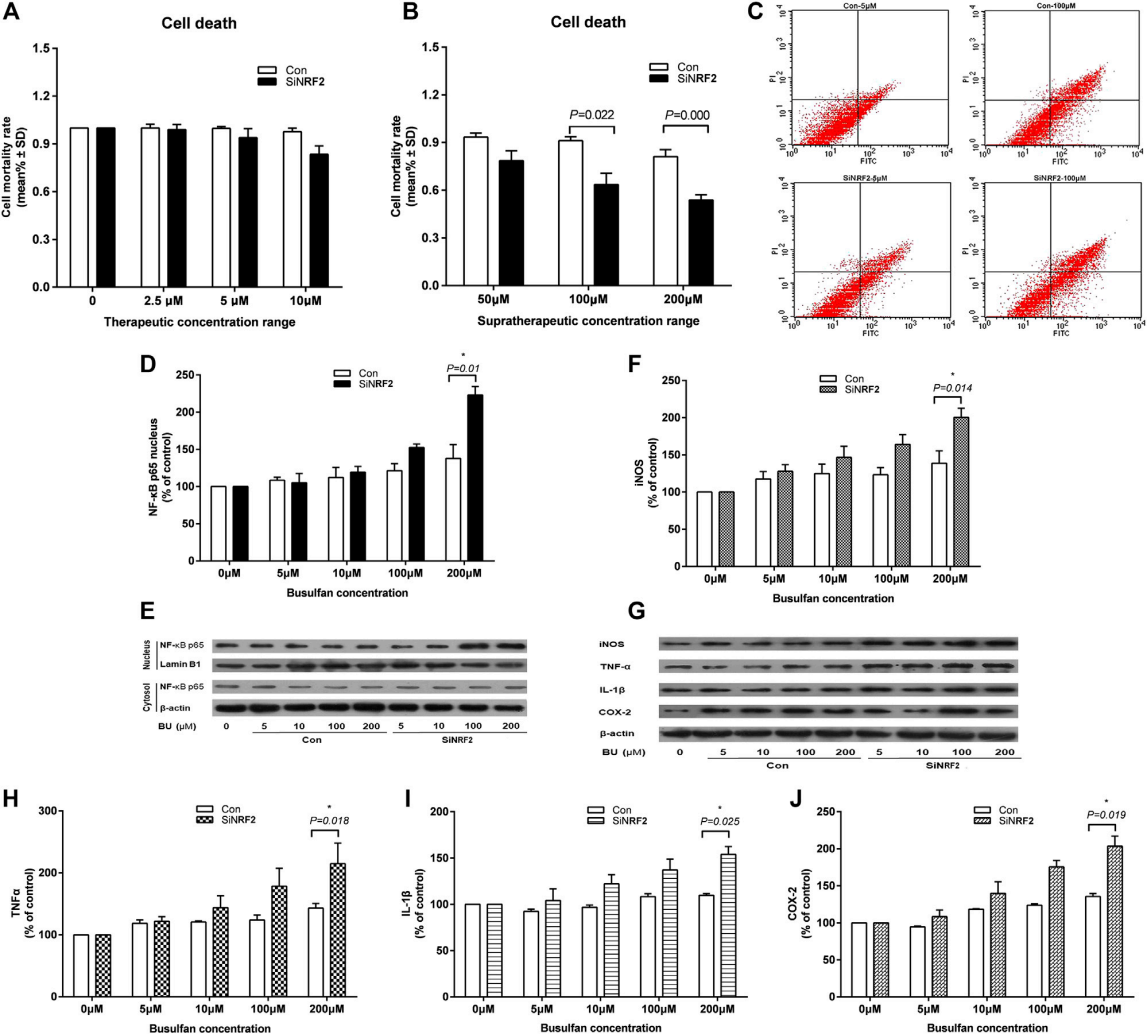
High exposure of endothelial cell to BU induced a proinflammatory response mediated by NF-κB and the toxicity in the absence of Nrf2. Death **(A** and **B)** and apoptosis **(C)** of human vascular endothelial cells (EA. hy 926) transfected with NRF2 specific siRNA (siNRF2) or not (Control) exposed to therapeutic or supratherapeutic BU concentrations. Western blot analysis of NF-κB **(D** and **E)**, iNOS, TNF-α, IL-1β, and COX-2 **(F** and **G, H, I, J)** proteins levels in EA. hy 926 cells exposed to increasing BU concentrations.

EA. hy 926 cells were treated with therapeutic BU at a concentration range of 2.5–10 μmol and a high concentration range of 50–200 μmol (Mei et al., 2014; Bouligand et al., 2016). The death of siNRF2-transfected cells exposed to high BU concentrations (*p* = 0.022 at 100 μmol and *p* < 0.001 at 200 μmol) significantly increased in comparison with that of control cells (Figures 4A–C). Consistently, the siNRF2 cells showed a dramatic elevation of nucleus NF-κB-p65 level at 200 µmol of BU (*p* = 0.010; Figures 4D,**E**). NF-κB hyperstimulation increased the production and secretion of iNOS, IL-1β, TNF-α, and COX-2 levels (Figures 4F–J).

#### BU Significantly Activated Nrf2 Pathway

**FIGURE 5:**
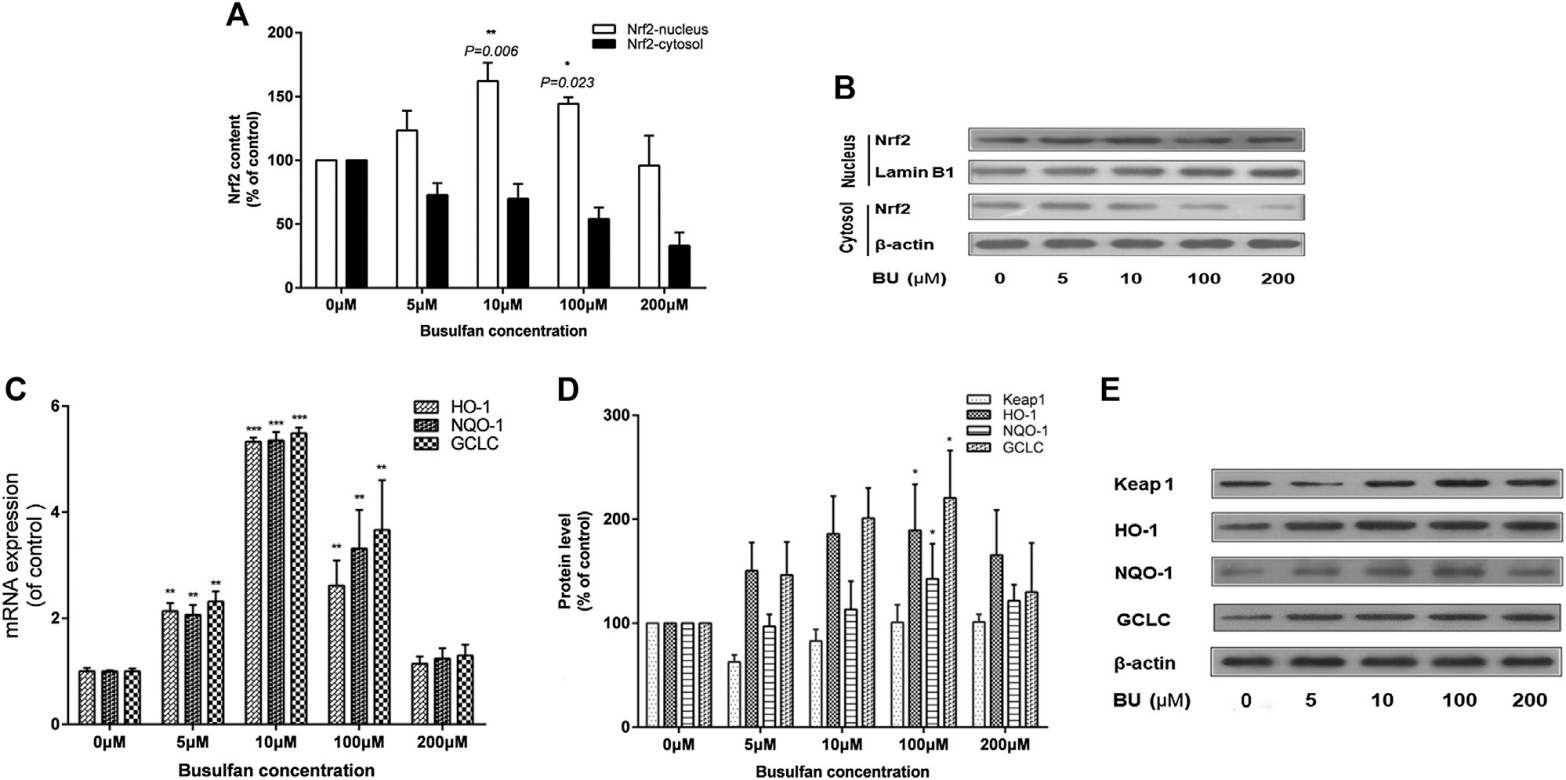
BU significantly activated Nrf2 signaling pathway. Nrf2 activation and its downstream proteins levels were gradually inhibited as the BU exposure increased significantly. Nrf2 protein levels (western blot analysis: **A, B)**, HO-1, NQO-1, and GCLC mRNA levels (real time PCR analysis: **C)** and their protein levels (western blot analysis: **D)** in EA. hy 926 cells exposed to the increasing BU concentrations. * represents *p* < 0.05; ** represents *p* < 0.01; *** represents *p* < 0.001; μM represents μmol.

Interestingly, Nrf2 was significantly activated by BU (*p* = 0.006 at 10 μmol; Figures 5A,B). BU exposure also increased the levels of HO-1, NQO-1, and GCLC mRNA and proteins (Figure 5C–E). However, the activation of Nrf2 and its downstream proteins was gradually inhibited as BU exposure of EC increased significantly.

## Discussion

In this study of HSCT patients, *NRF2 -617 CA/AA* genotypes, associated with high proinflammatory cytokine levels, predicted poor OS and increased TRM, especially for those with high BU AUC (>9.27 mg/L × h). *In vitro*, it was confirmed that high exposure of EC to BU, in the absence of Nrf2, induced a proinflammatory response mediated by NF-κB-p65 and led to EC death.

The *NRF2 -617CA/AA* patients receiving pretransplant BU-based conditioning regimens showed high plasma proinflammatory cytokine levels after HSCT. The study of Min et al., 2001 showed that the cytokines were involved in systemic inflammatory reactions and EC inflammation. A high level of cytokine IL-6, produced by almost all cells in response to any type of simulation, may be an early predictor of acute GVHD. Similarly, the increased level of cytokine TNF-α, which could induce EC injury and death, was observed in the plasma of GVHD patients. The systemic concentration of cytokine IL-8, abundantly produced by normal hepatocytes, can be elevated when EC injury in the liver occurs during SOS. The production of these proinflammatory cytokines (IL-6, IL-8, and TNF-α) could be suppressed by Nrf2, thereby alleviating the inflammatory response (Yang et al., 2018), while *NRF2 -617 CA/AA* mutation affects the positive feedback loop of transcriptional activation of the NRF2 and then significantly decreases the basal Nrf2 protein levels (Ishikawa, 2014). Therefore, *NRF2 -617CA/AA* patients with low Nrf2 expression may be vulnerable to inflammation response from high BU exposure.

We exhibited that *NRF2 -617CA/AA* genotypes could significantly impact TRM (HR = 4.04, *p* = 0.0142) and OS (HR = 3.69, *p* = 0.0272) in the patients with high BU AUC (>9.27 mg/L×h) compared to those with therapeutic AUC. Additionally, *NRF2 -617 CA/AA* genotypes were predictive of poor OS (RR = 3.91, *p* = 0.040) and high TRM (HR = 4.17, *p* = 0.041) in HSCT patients. The disease status was not a significant predictor for TRM or OS, which was not consistent with that of Bonifazi et al., 2014. That was probably attributed to the homogeneity of patients who mostly achieved their first CR before transplant. However, it was confirmed that high and subtherapeutic BU AUC, respectively, led to TRM and relapse (*p* = 0.048 and *p* = 0.005). These results were similar to those reported in the study of Bouligand et al., 2016. Recently, an increasing percentage of disease relapse has become the main cause of treatment failures in HSCT patients. Those patients who suffer relapse commonly face the risk of death from primary disease or receive second HSCT, resulting in an increasing medical and financial burden (Ma et al., 2017). In our enrolled patients, low BU AUC of 8.54 mg/L × h (95% CI: 8.06–9.02 mg/L × h) had 12 (13.8%) subtherapeutic AUCs (<7.38 mg/L × h) and only five supratherapeutic AUCs (>13.3 mg/L × h), which was basically consistent with the mean BU AUC (6.94 mg/L × h) of Chinese adult patients in another study (Yin et al., 2015). Our results showed that *NRF2 -617 CA/AA* genotypes may exacerbate the toxicity of high BU exposure. Hence, it may be necessary for the patients with high-risk hematologic malignancies to intensively monitor the dose-escalated BU, combined with *NRF2 -617CA/AA* genotyping, in order to avoid the possible drug toxicities induced by high exposure.

*In vitro*, high exposure of EC to BU, in the absence of Nrf2, elicited a significant EC injury and the hyperstimulation of NF-κB, accompanied with the elevated expressions of iNOS, IL-1β, TNF-α, and COX-2. Nrf2 activation could be inhibited as BU exposure of EC increased to a high level. Consistently, the downregulation of GCLC, HO-1, and NQO-1 indicated a decline of both GSH synthesis and antioxidative stress. Besides, the study of DeLeve and Wang, 2000 showed that the GSH-GSTs metabolism for BU depleted two GSH to the final metabolite, GSG. The decreased antioxidation of GSH-GSTs after BU treatment is commonly followed by various stimuli, e.g., other conditioning agents and microbial products, during HSCT, leading to EC injury. EC injury and interplay of inflammation are involved in the early pathophysiological process of the fatal toxicities of BU such as SOS and aGVHD, which cause inferior outcomes after HSCT (Vion et al., 2015). Furthermore, Nrf2 seemed to prevent EC injury under therapeutic exposure of BU via the crosstalk between Nrf2 and NF-κB. The P65 and Keap 1 may be the interactive proteins linking the Nrf2 and NF-κB pathways (Wardyn et al., 2015). However, the conditional and dynamic mechanism about the influence of low Nrf2 level, caused by *NRF2 -617CA/AA* genotypes, on NF-κB pathway via the crosstalk remains to be further studied.

This study is limited by its retrospective nature and small sample size. Additionally, with exception of pharmacokinetics interaction between BU and FLU, we did not find the other drug interactions of BU in clinic. However, there have been the inconsistent results of studies about the drug interactions among intravenous BU, CY, FLU, and oral phenytoin (De Jonge et al., 2005; Beumer et al., 2014; Myers et al., 2017). A large-scale and multicenter prospective study of HSCT patients without the potential risk of those drug interactions is warranted to clarify the influence of *NRF2 -617CA/AA* genotypes on early mortality after HSCT (100-day TRM), especially for those with supratherapeutic AUC (>13.3 mg/L × h). Due to the drawback of EC line, the impact of BU exposure on the expressions of GSTs and MRPs is needed to be validated in hepatocytes.

## Conclusion

NRF2 -617 CA/AA genotypes, associated with high proinflammatory cytokine levels, could predict inferior outcomes in HSCT patients with high BU AUC. *NRF2 -617 CA/AA* genotyping combined with TDM would optimize personalized BU dosing for sufficient efficacy and safety endpoint.

## Data Availability Statement

The raw data supporting the conclusions of this article will be made available by the authors, without undue reservation, to any qualified researcher.

## Ethics Statement

The studies involving human participants were reviewed and approved by Ruijin Hospital Research Ethics Committee. The patients/participants provided their written informed consent to participate in this study.

## Author Contributions

JH designed the study and wrote the manuscript. CH performed the study and wrote the manuscript. JH and WY regulated the entire project and reviewed the manuscript. LW, JJ, WT, and LW performed the study. ZL analyzed the data and interpreted the results. WZ provided the reagents and materials.

## Funding

This work was funded by the National Natural Science Foundation in China (No. 81503137).

## Conflict of Interest

The authors declare that the research was conducted in the absence of any commercial or financial relationships that could be construed as a potential conflict of interest.
