# Supplementary material for: NRF2 -617 C/A Polymorphism Impacts Proinflammatory Cytokine Levels, Survival, and Transplant-Related Mortality After Hematopoietic Stem Cell Transplantation in Adult Patients Receiving Busulfan-Based Conditioning Regimens
Source: Front Pharmacol. 2020 Dec 15;11:563321. doi: 10.3389/fphar.2020.563321 (PMC7770105; doi:10.3389/fphar.2020.563321)
Supplement: Supplementary file 1 [file table1.docx]

**Table S1 List of primers for SNaPshot analysis**

| **Genes** | **rs number** | **Forward primer** | **Reverse primers** |
| --- | --- | --- | --- |
| NRF2 | rs6706649_rs35652124_rs6721961 | CTCACTTTACCGCCCGAGAATG | CGCTTTGGTGGGAAGAGGTTCT |
| GSTP1 | rs1695 | CTCATCCTTCCACGCACATCCT | TTTCTTTGTTCAGCCCCCAGTG |
| GSTA1 | rs3957357 | GCTCGACAACTGAATTCCAGGTC | CCCTAGTCTTTGCACCCAACTCAT |
| GSTA1 | rs4715333 | TGCGTCACTCAAATTGCCCATA | CTGGGTCTGTGTTCCAGCACCT |
| GSTA1 | rs58912740 | GGGAAGGATTCCTGGAAATCACTT | TCCCTCAGTTTTGTAAGGGGTGA |
| GSTA1 | rs11964968 | CAAGCTGATGCCACCGTTTTCT | GCCAACATAACCCCCTACATGGT |
| GSTA2 | rs2180314 | CAAGGCAAGCTTGGCATCTTGT | TGGAGGTGAATTATTTTGCCATCAC |
| GCLM | rs41303970 | CTGGTGAGGTAGACACCGCCTC | GCAGTTTGGGAGAAGGTCCTTGA |
| GCLM | rs743119 | AGTTCAGCAGGTTCCCCGTCTG | CGCTCCCTCTCGGGTCTCTCT |
| GCLC | rs17883901 | CGATCCTGCGCTCCAGGTTTTA | TCAACACATTCTGCCGCTCTCA |
| MRP1 | rs4148356 | AGCAGGCCTGGATTCAGAATGA | TGACTGATTCAGGGGCCAACAT |
| MRP2 | rs2273697 | TTGGCTTTGTCCATGGGTCCTA | TTCTGGGCATCCACAGACATCA |
| MRP2 | rs3740066 | TGATAAGAGGCCTCCGCCAGAT | CCAGCTGCTCTCCACTCTGTCC |
| MRP2 | rs717620 | TGTTGGCCAGCTCTGTTGACAT | CATGATTCCTGGACTGCGTCTG |
