# Supplementary material for: NRF2 -617 C/A Polymorphism Impacts Proinflammatory Cytokine Levels, Survival, and Transplant-Related Mortality After Hematopoietic Stem Cell Transplantation in Adult Patients Receiving Busulfan-Based Conditioning Regimens
Source: Front Pharmacol. 2020 Dec 15;11:563321. doi: 10.3389/fphar.2020.563321 (PMC7770105; doi:10.3389/fphar.2020.563321)
Supplement: Supplementary file 2 [file table2.docx]

**Table S2 List of Primers for Real-time PCR**

| **Target gene** | **Forward primer** | **Reverse primers** |
| --- | --- | --- |
| GCLC | CAAGGACGTTCTCAAGTGG | TCTGGTCTCCAAAGGGTAG |
| HO-1 | AGGTATAGCAGATTTGGGTGAA | AAAGGCAGGGAAGTAGCG |
| NQO-1 | CATCCTGCGTCACCTGG | CTTGCCCGCCTCATAGTT |
| GAPDH | AAGGTCGGTGTGAACGGATTTG | TGTAGTTGAGGTCAATGAAGGGGTC |
