# Supplementary material for: NRF2 -617 C/A Polymorphism Impacts Proinflammatory Cytokine Levels, Survival, and Transplant-Related Mortality After Hematopoietic Stem Cell Transplantation in Adult Patients Receiving Busulfan-Based Conditioning Regimens
Source: Front Pharmacol. 2020 Dec 15;11:563321. doi: 10.3389/fphar.2020.563321 (PMC7770105; doi:10.3389/fphar.2020.563321)
Supplement: Supplementary file 3 [file table3.docx]

**Table S3 Genetic frequency distributions of the enrolled patients**

| **Genes** | **rs number** | **Nucleotide Change** | **MAF** | **Region** | **Protein** | **Genotypes, n (%)** | | | | | | **Alleles, %** | | | | **HWE(*P*)** |
| --- | --- | --- | --- | --- | --- | --- | --- | --- | --- | --- | --- | --- | --- | --- | --- | --- |
| NRF2 | rs6706649 | C>T | 0.063 | upstream_transcript |  | CC | 75(86.2) | CT | 12(13.8) | TT | 0(0) | C | 0.931 | T | 0.069 | 0.49 |
| NRF2 | rs6721961 | G>T | 0.243 | upstream_transcript |  | GG | 50(57.5) | TG | 35(40.2) | TT | 2(2.3) | G | 0.776 | T | 0.224 | 0.14 |
| NRF2 | rs35652124 | T>C | 0.552 | upstream_transcript |  | TT | 25(28.7) | TC | 44(50.6) | CC | 18(20.7) | T | 0.540 | C | 0.460 | 0.87 |
| GSTP1 | rs1695 | A>G | 0.179 | coding | Ile 105 Val | AA | 60(69.0) | AG | 23(26.4) | GG | 4(4.6) | A | 0.822 | G | 0.178 | 0.36 |
| GSTA1 | rs3957357 | G>A | 0.139 | 5' UTR |  | GG | 65(74.7) | AG | 21(24.2) | AA | 1(1.1) | G | 0.868 | A | 0.132 | 0.63 |
| GSTA1 | rs4715333 | A>C | 0.367 | upstream_transcript |  | AA | 47(54.0) | CA | 35(40.3) | CC | 5(5.7) | A | 0.694 | C | 0.306 | 0.16 |
| GSTA1 | rs58912740 | G>T | 0.137 | upstream_transcript |  | GG | 65(74.7) | GT | 21(24.1) | TT | 1(1.1) | G | 0.868 | T | 0.132 | 0.63 |
| GSTA1 | rs11964968 | T>C | 0.132 | upstream_transcript |  | TT | 63(72.4) | TC | 22(25.3) | CC | 2(2.3) | T | 0.851 | C | 0.149 | 0.96 |
| GSTA2 | rs2180314 | G>C | 0.315 | coding | Ser112Thr | GG | 42(48.8) | CG | 40(45.9) | CC | 5(5.8) | G | 0.715 | C | 0.285 | 0.29 |
| GCLM | rs41303970 | G>A | 0.164 | 5' UTR |  | GG | 69(79.3) | AG | 17(19.5) | AA | 1(1.2) | G | 0.891 | A | 0.109 | 0.97 |
| GCLM | rs743119 | C>A | 0.163 | 5' UTR |  | CC | 69(79.3) | CA | 18(20.7) | AA | 0(0) | C | 0.897 | A | 0.103 | 0.28 |
| GCLC | rs17883901 | G>A | 0.134 | upstream_transcript |  | GG | 69(79.3) | AG | 16(18.4) | AA | 2(2.3) | G | 0.885 | A | 0.115 | 0.37 |
| MRP1 | rs4148356 | G>A | 0.048 | coding/intron | Arg723Gln | GG | 80(92) | AG | 7(8) | AA | 0(0) | G | 0.960 | A | 0.040 | 0.70 |
| MRP2 | rs2273697 | G>A | 0.096 | coding | Val417Ile | GG | 76(87.4) | AG | 10(11.5) | AA | 1(1.1) | G | 0.931 | A | 0.069 | 0.33 |
| MRP2 | rs3740066 | C>T | 0.247 | downstream_transcript |  | CC | 53(60.9) | CT | 29(33.3) | TT | 5(5.7) | C | 0.776 | T | 0.224 | 0.70 |
| MRP2 | rs717620 | C>T | 0.217 | 5' UTR |  | CC | 54(62.1) | CT | 28(32.2) | TT | 5(5.7) | C | 0.782 | T | 0.218 | 0.59 |

MAF: minor allele frequency in East Asians; HWE: Hardy-Weinberg equilibriu
