# Supplementary material for: NRF2 -617 C/A Polymorphism Impacts Proinflammatory Cytokine Levels, Survival, and Transplant-Related Mortality After Hematopoietic Stem Cell Transplantation in Adult Patients Receiving Busulfan-Based Conditioning Regimens
Source: Front Pharmacol. 2020 Dec 15;11:563321. doi: 10.3389/fphar.2020.563321 (PMC7770105; doi:10.3389/fphar.2020.563321)
Supplement: Supplementary file 4 [file table4.docx]

**Table S4 Influence of the involved genetic polymorphisms, conditionings, and busulfan AUC on plasma IL-6, IL-8, and TNF-ɑ levels**

| **Variables** | **Categories** | **IL-6** | | |  | **IL-8** | | |  | **TNF-ɑ** | | |
| --- | --- | --- | --- | --- | --- | --- | --- | --- | --- | --- | --- | --- |
|  |  | **mean** | **F** | **P** |  | **mean** | **F** | **P** |  | **mean** | **F** | **P** |
| NRF2-651 G/A | GG | 22.2±13.4 | 1.166 | 0.288 |  | 54.1±42.8 | 1.917 | 0.175 |  | 6.26±1.88 | 3.422 | 0.72 |
|  | GA/AA | 18.1±9.41 |  |  |  | 39.1±15.6 |  |  |  | 7.74±2.82 |  |  |
| NRF2-617 C/A | CC | 22.1±10.2 | 27.799 | <0.001 |  | 35.3±9.45 | 49.309 | <0.001 |  | 8.26±2.50 | 20.083 | <0.001 |
|  | CA/AA | 9.12±3.21 |  |  |  | 18.6±4.35 |  |  |  | 5.50±0.96 |  |  |
| NRF2-653 A/G | AA | 24.4±11.7 | 1.105 | 0.301 |  | 43.2±19.6 | 0.504 | 0.483 |  | 6.95±1.93 | 2.975 | 0.09 |
|  | AG/GG | 19.0±18.4 |  |  |  | 50.4±38.5 |  |  |  | 5.77±2.15 |  |  |
| GSTP1 I105V | *A*A | 23.2±17.0 | 1.412 | 0.242 |  | 50.5±37.6 | 1.677 | 0.204 |  | 7.19±4.01 | 0.479 | 0.473 |
|  | *A *B/*B *B | 17.9±8.95 |  |  |  | 38.5±15.0 |  |  |  | 7.91±2.14 |  |  |
| GSTA1-69 C/T | *A*A | 24.0±18.3 | 1.32 | 0.258 |  | 54.9±45.7 | 2.438 | 0.127 |  | 6.13±1.67 | 2.958 | 0.094 |
|  | *A *B/*B *B | 18.7±8.40 |  |  |  | 37.7±14.6 |  |  |  | 7.18±2.09 |  |  |
| GSTA2 S112T | ser/ser | 16.6±7.69 | 1.458 | 0.235 |  | 34.4±12.9 | 1.722 | 0.198 |  | 7.30±2.36 | 0.013 | 0.908 |
|  | thr+ | 21.4±15.5 |  |  |  | 40.6±15.9 |  |  |  | 7.21±2.56 |  |  |
| GCLC-129C/T | CC | 18.4±8.39 | 0.048 | 0.828 |  | 43.1±12.8 | 0.659 | 0.422 |  | 7.20±1.70 | 2.743 | 0.106 |
|  | CT/TT | 17.6±12.1 |  |  |  | 51.0±40.5 |  |  |  | 6.38±1.34 |  |  |
| GCLM-588C/T | CC | 18.7±8.29 | 1.677 | 0.204 |  | 47.2±44.6 | 0.784 | 0.382 |  | 6.33±2.48 | 1.342 | 0.254 |
|  | CT/TT | 15.6±6.50 |  |  |  | 36.7±12.6 |  |  |  | 7.13±1.71 |  |  |
| GCLM-23G/T | GG | 17.6±10.4 | 1.419 | 0.241 |  | 40.0±19.5 | 1.636 | 0.209 |  | 6.26±1.76 | 2.071 | 0.159 |
|  | GT/TT | 21.9±12.1 |  |  |  | 51.6±34.2 |  |  |  | 7.40±2.96 |  |  |
| Conditionings | BU/CY | 17.8±9.82 | 2.294 | 0.139 |  | 41.8±29.5 | 0.16 | 0.692 |  | 6.60±2.44 | 1.452 | 0.236 |
|  | BU/FLU | 24.7±18.0 |  |  |  | 45.3±24.2 |  |  |  | 7.55±2.46 |  |  |
| BU AUC | Sub-therapeutic | 18.6±11.1 | 3.789 | 0.058 |  | 33.0±18.3 | 3.095 | 0.086 |  | 5.87±2.20 | 4.521 | 0.151 |
|  | Above-therapeutic | 12.8±10.0 |  |  |  | 43.7±22.1 |  |  |  | 6.76±1.99 |  |  |
