# Supplementary material for: NRF2 -617 C/A Polymorphism Impacts Proinflammatory Cytokine Levels, Survival, and Transplant-Related Mortality After Hematopoietic Stem Cell Transplantation in Adult Patients Receiving Busulfan-Based Conditioning Regimens
Source: Front Pharmacol. 2020 Dec 15;11:563321. doi: 10.3389/fphar.2020.563321 (PMC7770105; doi:10.3389/fphar.2020.563321)
Supplement: Supplementary file 5 [file table5.docx]

**Table S5 Univariate analysis of the involved genetic polymorphisms on relapse, TRM, and OS**

| **Variables** | **rs number** | **TRM^#^** | **OS** | **Relapse** |
| --- | --- | --- | --- | --- |
|  |  | ***P*** | ***P*** | ***P*** |
| NRF2-653 A/G | rs6706649 | 0.993 | 0.984 | 0.644 |
| NRF2-617 C/A | rs6721961 | **0.038** | **0.044** | 0.272 |
| NRF2-651 G/A | rs35652124 | 0.125 | 0.129 | 0.184 |
| GSTP1 I105V | rs1695 | 0.567 | 0.792 | 0.844 |
| GSTA1-69 C/T | rs3957357 | 0.084 | 0.313 | 0.205 |
| GSTA2 S112T | rs2180314 | 0.251 | 0.288 | 0.424 |
| GCLM-588 C/T | rs41303970 | 0.557 | 0.805 | 0.663 |
| GCLM-23 G/T | rs743119 | 0.557 | 0.871 | 0.377 |
| GCLC-129 C/T | rs17883901 | 0.289 | 0.549 | 0.774 |
| MRP1-2168 G/A | rs4148356 | 0.839 | 0.95 | 0.318 |
| MRP2-3972 C/T | rs3740066 | 0.237 | 0.777 | 0.246 |
| MRP2-1249 G/A | rs2273697 | 0.252 | 0.653 | 0.968 |
| MRP2-24 C/T | rs717620 | 0.243 | 0.668 | 0.651 |

^#^The competing risk analysis was used to analyze the influence of genetic polymorphisms on TRM; OS, overall survival; TRM, transplant-related mortality.
