# Supplementary figures and images for: NRF2 -617 C/A Polymorphism Impacts Proinflammatory Cytokine Levels, Survival, and Transplant-Related Mortality After Hematopoietic Stem Cell Transplantation in Adult Patients Receiving Busulfan-Based Conditioning Regimens
Source: Front Pharmacol. 2020 Dec 15;11:563321. doi: 10.3389/fphar.2020.563321 (PMC7770105; doi:10.3389/fphar.2020.563321)

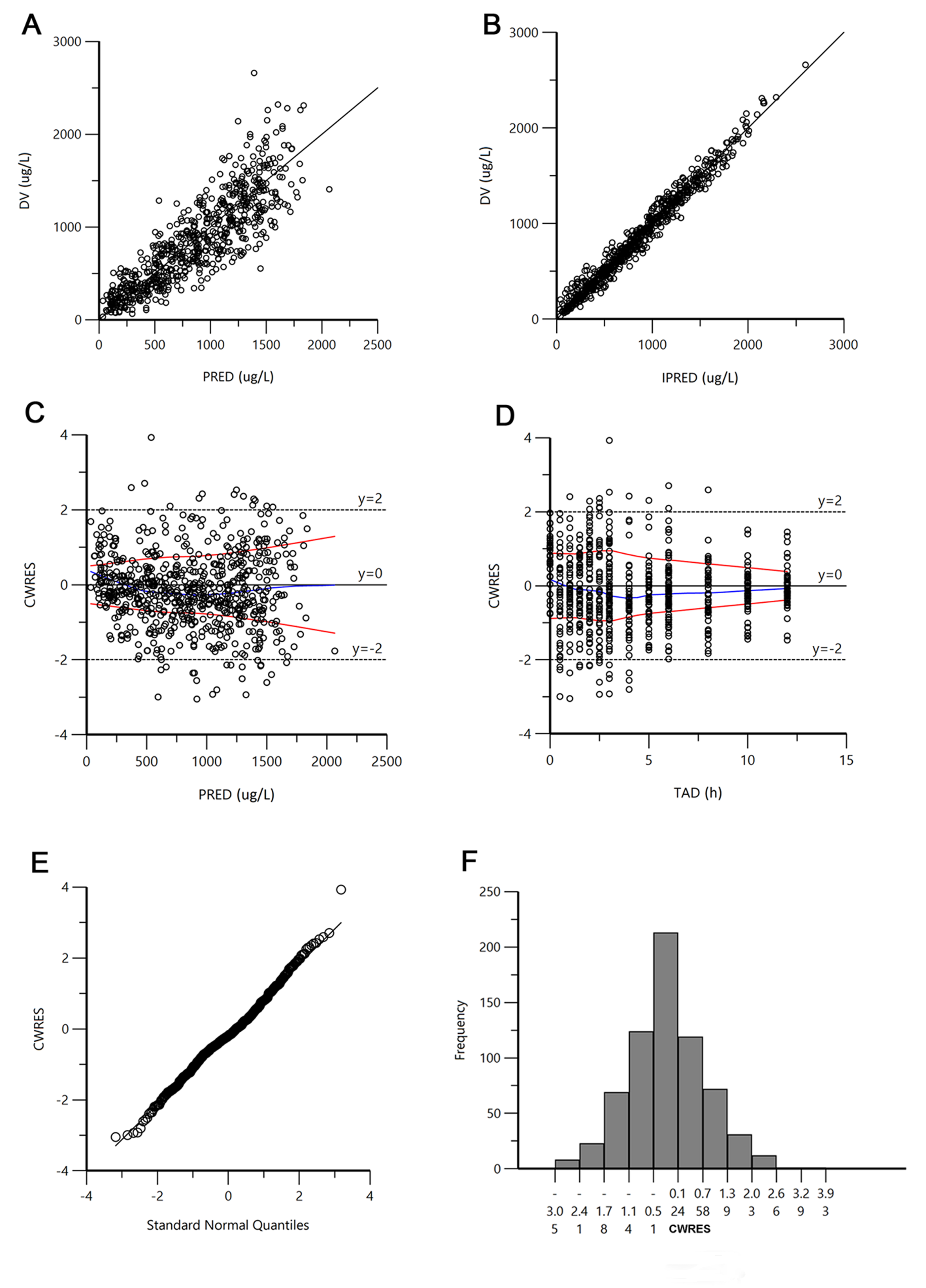

Supplement: Supplementary file 7 [file image1.tif]

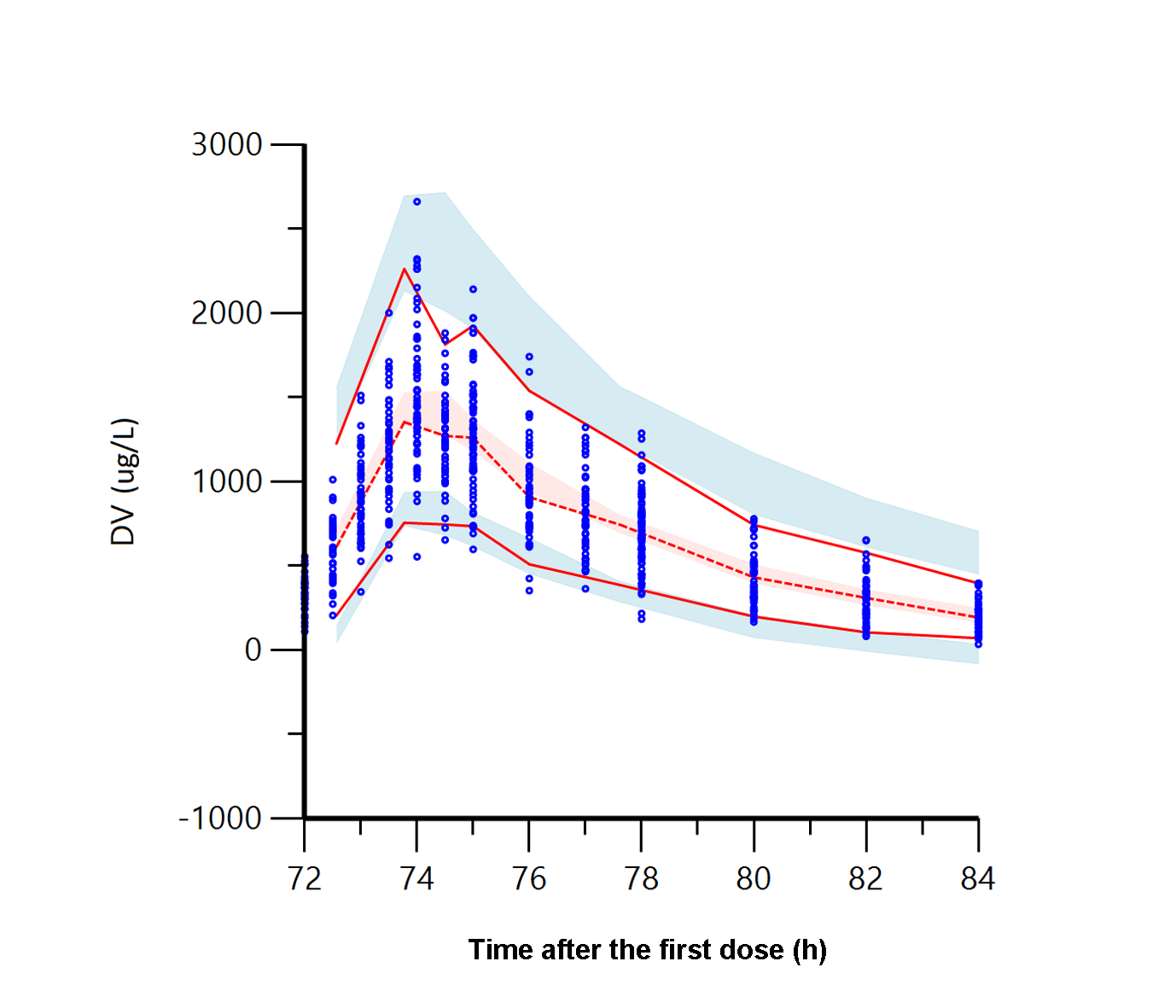

Supplement: Supplementary file 8 [file image2.tiff]
